# Predicting the Surface Tension of Deep Eutectic Solvents: A Step Forward in the Use of Greener Solvents

**DOI:** 10.3390/molecules27154896

**Published:** 2022-07-31

**Authors:** Amit Kumar Halder, Reza Haghbakhsh, Iuliia V. Voroshylova, Ana Rita C. Duarte, Maria Natalia D. S. Cordeiro

**Affiliations:** 1 LAQV@REQUIMTE, Department of Chemistry and Biochemistry, Faculty of Sciences, University of Porto, 4169-007 Porto, Portugal; up501129@g.uporto.pt; 2 Dr B C, Roy College of Pharmacy and Allied Health Sciences, Dr. Meghnad Saha Sarani, Bidhannagar, Durgapur 713212, WB, India; 3 Department of Chemical Engineering, Faculty of Engineering, University of Isfahan, Isfahan 81746-73441, Iran; haghbakhsh@gmail.com; 4 LAQV@REQUIMTE, Department of Chemistry, NOVA School of Science and Technology, 2829-516 Caparica, Portugal; ard08968@fct.unl.pt

**Keywords:** DES, surface tension, in silico-based models, QSPR, validation, consensus modeling

## Abstract

Deep eutectic solvents (DES) are an important class of green solvents that have been developed as an alternative to toxic solvents. However, the large-scale industrial application of DESs requires fine-tuning their physicochemical properties. Among others, surface tension is one of such properties that have to be considered while designing novel DESs. In this work, we present the results of a detailed evaluation of Quantitative Structure-Property Relationships (QSPR) modeling efforts designed to predict the surface tension of DESs, following the Organization for Economic Co-operation and Development (OECD) guidelines. The data set used comprises a large number of structurally diverse binary DESs and the models were built systematically through rigorous validation methods, including ‘mixtures-out’- and ‘compounds-out’-based data splitting. The most predictive individual QSPR model found is shown to be statistically robust, besides providing valuable information about the structural and physicochemical features responsible for the surface tension of DESs. Furthermore, the intelligent consensus prediction strategy applied to multiple predictive models led to consensus models with similar statistical robustness to the individual QSPR model. The benefits of the present work stand out also from its reproducibility since it relies on fully specified computational procedures and on publicly available tools. Finally, our results not only guide the future design and screening of novel DESs with a desirable surface tension but also lays out strategies for efficiently setting up silico-based models for binary mixtures.

## 1. Introduction

The last two decades have witnessed a significant shift in the design and development of new chemicals for large-scale industrial applications. One of such efforts has been driven towards the replacement of flammable and environmentally hazardous substances with green and sustainable solvents. More benign solvents, even if dispensed in a large amount into the environment, are known to produce less harmful effects [1,2,3]. Deep eutectic solvents (DES) represent a class of “green solvents” with tremendous potential to replace conventional toxic chemicals. Indeed, apart from having a wide range of applications, DESs exhibit much less environmental toxicity, even when compared to their predecessor, ionic liquids (ILs) [4,5]. Thus, it is not surprising that the emergence of DESs at the beginning of this century drew considerable attention from the scientific community, as is confirmed by the growing number of publications related to DESs in the last two decades [4,5]. DESs may simply be defined as the low melting point mixture of at least two compounds, one acting as a hydrogen bond acceptor (HBA) and another as a hydrogen bond donor (HBD) in a specific molar ratio [6,7]. Their low melting point, which is a result of complex hydrogen bonding interactions between the components of DESs, allows them to remain in the liquid phase at room temperature [8]. Besides being less eco-toxic in nature, DESs are generally easy to prepare, cost-effective and biocompatible [7,8,9]. However, even with many advantages, the suitability of any chemical for long-term industrial applications often depends on its fundamental physical properties, such as density, viscosity, surface tension, vapor pressure, the speed of sound etc. [4,5], and DESs are of no exception. The physicochemical profile of a DES can readily be tailored by choosing different combinations of their starting components or by modifying their chemical structures [10,11]. Still, the number of possible combinations that can be envisaged to form DESs is extremely high. As such, without detailed knowledge of the relation between structure and properties, their fine-tuning is barely applicable in practice and often limited to a trial-and-error procedure.

Surface tension is one of the most crucial physical properties that must be considered, as it is required for the set-up of industrial processes, such as the design of heating systems, distillation columns and heat exchangers [12]. Therefore, the measurement of their surface tension is essential to assess the suitability of DESs for industrial applications. Normally, with increasing temperature, the surface tension of a DES decreases, but it is well-known that its components and their molar ratio are also responsible for their resulting surface tension [13].

Previously, we have reported a general thermodynamic model to estimate the surface tension for DESs of different nature [14]. The model has been developed with an up-to-date data bank containing surface tension values for a large number of structurally diverse DESs. The question that yet remains is whether a more predictive model for the surface tension of DESs can be achieved by alternative in silico-based modeling approaches such as the one proposed here, Quantitative Structure-Property Relationships (QSPR). In fact, the application of QSPR modeling techniques has long stood as particularly useful to estimate a wide range of properties of different materials [15,16,17,18,19,20,21]. Thus, many QSPR modeling studies have been devoted to different physicochemical properties of DESs. Very recently, for example, Wang et al. developed QSPR models based on Conductor-like Screening Model for Real Solvents (COSMO-RS) descriptors for characterizing the CO_2_ solubility in DESs [22]. The authors found that the linear model was unable to successfully fit the whole dataset but a random forest non-linear model showed greater reliability, judging from the Absolute-Average-Relative-Deviation (AARD) value of 7.8% attained for that data (59 DESs). Balali and co-workers developed also QSPR models for probing the thiophene distribution between choline chloride (ChCl)-based DES and hydrocarbon phases in ternary systems [23]. The proposed linear models displayed good accuracy and included topological descriptors, which indicate the influence of the degree of the structure of HBDs on the thiophene distribution. In another study, Khajeh et al. employed QSPR modeling for predicting the melting and freezing points of DESs [24]. Their results showed that both properties of 181 DESs could be predicted with good accuracy (*R*^2^~0.80) by the derived linear models. Multiple attempts have been undertaken as well to set up linear and non-linear QSPR-based models for estimating the density and viscosity of DESs [25,26,27]. All the later models resorted to COSMO-RS descriptors and showed great predictivity performance (*R*^2^ > 0.95). However, these models have been based on a small number of data points pertaining to just certain families of DESs (e.g.: 49 hydrophilic [26] or 54 hydrophobic [27] DESs), which thus limits their general applicability.

The present work is encouraged by our very recent QSPR modeling efforts on the density of DESs [28], which yielded statistically more robust models than a thermodynamic model developed with the same dataset [29]. On one hand, such QSPR efforts demonstrated to offer more options and versatility for setting up predictive models as compared to thermodynamic modeling. On the other hand, QSPR models demand several statistical conditions be satisfied just as inspected here as per the guidelines of the Organization for Economic Co-operation and Development (OECD) [30] to expand their overall applicability as well as statistical reliability [31]. Moreover, in order to address the requirement of robust validation strategies applicable for the binary mixtures, we have recently designed an open-source standalone Python-based tool “QSAR-Mx” (freely available to download at https://github.com/ncordeirfcup/QSAR-Mx, last accessed on 28 April 2022) [28]. This work extensively utilizes such a tool to set up the predictive QSPR models for probing the surface tensions of DESs. Therefore, the scope of the current work goes beyond the development of QSPR models by proposing and comparatively testing novel methodologies that can be utilized in the future towards reliably predicting the properties of binary mixtures.

## 2. Materials and Methods

### 2.1. Dataset Collection and Splitting

The dataset employed for the development of the QSPR models was adopted from our previously published work on DES surface tension estimation [14]. It contains 553 data points, compiled from 112 different binary and ternary DESs of diverse compositions. However, here we solely focused on binary DESs and therefore the current dataset was reduced to 530 data points pertaining to 99 different binary DESs. This dataset was combined with an additional set comprising 89 data points that were collected from measurements reported in the literature since 2020 [32,33,34,35] and that, thus, were not included in our previously thermodynamic model. The final dataset comprises 619 unique data points coming from 113 different types of binary DESs. It is worth noting that the experimental surface tension of each DES in the dataset was measured at atmospheric pressure in a large temperature range (278.15–358.15 K), rendering the temperature an important independent variable to consider in the QSPR modeling for understanding how it influences such physical property.

Predictive validation is a required but delicate task in any QSPR modeling—i.e., to assess model adequacy to new mixtures, and it is related directly to the dataset division scheme adopted. In fact, as shown by Muratov et al. [36], the random division of the original dataset for validation purposes is unacceptable since it can lead to unreliable QSPR models and to an over-optimistic estimation of their predictive performance. The authors have thus proposed and described in detail different validation strategies for the QSPR modeling of mixtures [36]. In this work, two such validation strategies were utilized to search for the most predictive QSPR models, namely: the mixtures-out (MO) and compounds-out (CO) schemes. Briefly, in the MO scheme, mixtures of the modeling set are distributed among the training and the test sets without repetition. By contrast, in the CO scheme, at least one chemical of the dataset is never placed in the training set. Naturally, these validation strategies are only applicable to binary mixtures and require some guidance to follow. Due to the complexity of the data matrices, any random MO- or CO-based division scheme may not yield the most predictive model since variables selection depends largely on the training set [28]. Even though the CO-based validation is considered to be the most robust strategy [36], it may give rise to underfitted models with poor statistical quality. At the same time, while the MO-based validation is less robust, this strategy definitely provides more meaningful solutions than any random data distributions or other validation division schemes such as the points-out one proposed by the same authors [28,36,37,38].

As referred to above, we have recently developed a Python-based tool named QSAR-Mx [28], specifically devised to address and automate some crucial steps involved in the QSPR modeling of binary mixtures. A detailed description of the functionalities of this tool can be found in its instruction manual (accessible from https://github.com/ncordeirfcup/QSAR-Mx, last accessed on 28 April 2022). Essentially, QSAR-Mx lets the user generate multiple MO- and CO-based data distributions and then develop models with the latter and select the most predictive ones based on their statistical metrics. Firstly, the user should choose two parameters—i.e., seed and interval, for generating the MO- and CO-based data distributions. In the MO division scheme, QSAR-Mx detects unique mixtures present in the dataset and then sorts them by the number of occurrences in descending order. From the sorted list, the mixtures are grouped according to the seed (the starting point for selection) and interval values given. The unique mixtures selected are then incorporated into the test set. Likewise, in the CO scheme, the QSAR-Mx tool begins by sorting the unique chemicals that belong to component-1 of the mixtures, followed by sorting them in descending order and lastly, by choosing some chemicals based upon the maximum values of the seed and interval chosen. This procedure is then replicated for the unique chemicals belonging to component-2 of the mixtures. The unique chemicals selected are then placed in the test set. One should notice however here that the following QSPR models were always derived with generated data distributions in which the training set size was always greater than the test set size and, simultaneously, the size of the latter was at least 15% of the former. It should be also mentioned that the QSAR-Mx tool has been slightly modified since our previous work [28] because we found that the MO-based data distributions vary from one run to another. The new version of QSAR-Mx is now able to generate the same data distributions (i.e., MO-based training and test sets) every time, independently of the seed and interval given, leading thus to more reproducible modeling results.

In this work, to begin with, we divided the whole dataset into a modeling set and an external validation set (535 and 84 data points, respectively), using the CO-based division scheme with values for the seed and interval of 3 and 4, respectively. The modeling dataset was subsequently divided into training and test sets by MO- and CO-based schemes, setting both the maximum seed and maximum interval as 6. The DESs in the training set coming from the two schemes were employed separately for the development of the QSPR models, and those from the test sets were only used to test such models. The DESs in the external validation set were utilized for extra validation of the final most predictive QSPR models found. Details about the investigated DESs along with their experimental surface tension values, and corresponding references are given in Table S1 of the Supplementary Materials.

### 2.2. Mixture Descriptors

Due to the unique nature of binary DESs, the calculation of their descriptors requires additional steps to take into account the specificity of each component as well as the molar fractions. Here, we resorted to the strategy previously suggested by Oprisiu et al. [37], in which the descriptors are initially calculated for each component and then modified on the basis of ‘mixture descriptors weighted by molar fraction’ formulas. As such, two types of modified descriptors (from now on, referred to as WM descriptors), i.e., *D*_pmix_ and *D*_nmix_, were computed by the following formulas:

*D*_pmix_ = *x*_1_ *D*_1_ + *x*_2_ *D*_2_ (1)

*D*_nmix_ = |*x*_1_ *D*_1_ − *x*_2_ *D*_2_| (2)

where *D_i_* stands for the descriptor of each component *i* (*i* = 1, 2) and *x_i_* for the respective molar fraction in the mixture.

Both formulas have already been successfully applied to generate predictive models for various properties of DESs [9,28,38] and implemented in the QSAR-Mx tool. Basically, QSAR-Mx includes two methods for calculating such mixture descriptors. Starting from the descriptors previously obtained for each component (*D*_1_ and *D*_2_), ‘Method-1’ calculates only the *D*_pmix_ descriptors whereas ‘Method-2’ provides both the *D*_pmix_ and *D*_nmix_ descriptors. In the present work, we used both of the aforementioned methods separately and then performed a comparative analysis to elucidate the method that provides the best solution as far as the predictivity of the overall model is concerned.

To start with, the 3D structures of each DES component were obtained by inputting the SMILES (Simplified Molecular Input Line Entry Specification) strings into the application MarvinView (https://docs.chemaxon.com/display/docs/marvinview.md, last accessed on 15 March 2022) and subsequently standardized by the ChemAxon Standardizer tool with the following options: strip salts, aromatize, neutralize and add explicit hydrogen atoms [39]. Here, we have resorted to the 0D-2D descriptors available in the Dragon software [40] for describing each DES component. Actually, 3D descriptors were excluded due to the high computational effort required for structure optimization of each component, especially for large datasets, and the fact that those may also give rise to misleading information not ensuring reliable property prediction by 3D QSPR [28,41]. Finally, along with the WM descriptors, three independent variables were also included, namely: the measuring temperature, *T* (in K), the presence/absence of chlorine ions, and the presence/absence of bromine ions. The importance of temperature for the modeling was referred to before. Note in addition that only the cationic part was considered during the calculation of WM descriptors for the DES HBA component. Hence, two binary indicator variables (i.e., the presence (1) or absence (0) of halide ions) were required to be included to account for the anionic part of the HBA components.

### 2.3. Modeling Techniques and Evaluation

As to the modeling techniques, we started by opting for a regression-based approach like in our previous work [28] thanks to its easy interpretation but also high reliability. Specifically, the regression coefficients were obtained by the multiple linear regression analysis (MLR) implemented in our in-house QSAR-Mx tool and by selecting the variables through the sequential forward selection (SFS) algorithm using the Sequential Feature Selector module of Mlxtend (http://rasbt.github.io/mlxtend/) [42]. The following different conditions were applied for scoring the SFS selection:

(a) determination coefficient (*R^2^*), no cross-validation;
(b) negative mean absolute error (NMAE), no cross-validation;
(c) negative mean Poisson deviance (NMPD), no cross-validation;
(d) determination coefficient (*R*^2^), five-fold cross-validation (CV) or ten-fold CV.

Yet, a correlation cutoff of 0.95 and variance cutoff of 0.001 were always set to discard highly intercorrelated and near-constant descriptors. Additionally, the selection of the optimal number of descriptors for the MLR models was controlled by the %MAE_LOO_ reduction policy, also implemented in QSAR-Mx. The %MAE_LOO_ reduction scheme guarantees that no new descriptor is added to the model during feature selection if its inclusion does not reduce the leave-one-out (LOO) cross-validation and mean absolute error (MAE_LOO_) by at least 5% of the previous model. As such, this policy guarantees that the optimal number of descriptors is present in the model and, at the same time, that models generated with multiple model development strategies may be compared from a neutral ground [28].

To check if higher accuracy could be achieved when estimating the surface tension of DESs, non-linear models were also developed using five different machine learning (ML) techniques, i.e.: (i) *k*-Nearest Neighbors (*k*-NN), (ii) Random Forests (RF), (iii) Support Vector Machines (SVM), (iv) Neural Network Multilayer Perceptron (NN-MLP), and (v) Gradient boosting (GB) [43,44,45,46,47]. Such ML-based models were set up by resorting to the tools available in the Scikit-learn programs (https://scikit-learn.org/stable/) with QSAR-Mx (last accessed on 28 April 2022) and for each of them, hyperparameter tuning was performed by varying their crucial parameters (see the list in Table S2). The best parameters for a given ML estimator were determined by a 5-fold cross-validation scheme using the same training sets as before. In the same manner, the external predictivity of the promising non-linear models found was firstly accessed and further validated using the same test and external sets.

As described above, QSAR-Mx generates multiple models based on three types of inputs provided by the user, namely: (i) descriptor calculation strategy (Method-1 or -2), (ii) dataset division schemes (MO- or CO-based data-division), and (iii) scoring conditions. No matter the model generation strategy followed, any QSPR regression model requires an evaluation with robust diagnostic tools to assess and compare its acceptability as well as quality over other models.

In this work, the internal predictivity of the developed QSPR regression models was primarily checked by statistical parameters, such as the MAE_LOO_ and *Q*^2^_LOO_ (LOO cross-validation *R*^2^) [48,49]. Keeping in mind the importance of the compounds-out validation, we have recently introduced two new statistical parameters based on the so-called leave-chemical-out (LCO) cross-validation, which is conceptually similar to the well-known leave-many-out CV but more effective whenever dealing with mixtures [28]. These new parameters, i.e., *Q*^2^_LCO_ and MAE_LCO_, are mostly important for the MO-based data distributions. Indeed, even though the latter often produce more predictive models than the CO-based data distributions, their predictivity remains questionable due to the lack of CO-based validation. Both *Q*^2^_LCO_ and MAE_LCO_ have actually helped us monitor the model performance upon the removal of each component (belonging either to HBA or to HBD) one by one from the training set with further model redevelopment using the remaining components. A detailed description of these two parameters can be found in our previous work [28]. Importantly, while assessing the quality of the models, the difference between *Q*^2^_LOO_ and *Q*^2^_LCO_ should also be evaluated. In fact, a large discrepancy between the values of the latter suggests that the mixtures based on one or more components are not predicted well enough by the QSPR model. Besides the above-mentioned statistics, the internal predictivity of the final regression models was also evaluated by using scaled *r_m_*^2^ validation metrics, such as *r_m_*^2^_(LOO)_ and Δ*r_m_*^2^_(LOO)_ [50]. Basically, *r_m_*^2^ metrics are based on the correlation between the observed and predicted values, with and without setting to zero the intercept of the least square regression lines. In addition, the AARD calculated for each data distribution was also used for checking the overall errors of the derived models. Although not quite common in QSPR modeling, the latter allowed us to compare the quality of our QSPR models with that of previously reported thermodynamic models [29]. To access the external predictive ability of the models, similar statistical validation metrics were also employed, i.e., the mean absolute error for the test set or external validation set (MAE_test_ and MAE_ext_) and the variance explained for external prediction (*R*^2^_Pred_) [44] along with the scaled *r_m_*^2^_(test)_, Δ*r_m_*^2^_(test)_, *r_m_*^2^_(ext)_, and Δ*r_m_*^2^_(ext)_ metrics [50].

Other aspects that deserve special attention are the absence of highly collinear descriptors and the lack of chance correlations in the final derived models. Highly collinear variables were simply checked by inspecting the cross-correlation matrix of the models’ descriptors. On the other hand, the *Y*-randomization technique identifies models with chance correlations, using the *cR_P_*^2^ parameter [51], after the sequence of the response vector has been randomly modified. Here, the procedure was repeated 1000 times, and new models were developed with the randomly reordered responses employing the same set of variables. The uniqueness of the final regression model and its lack of chance correlations is confirmed by the value obtained for *cR_P_*^2^, which should be closer to one [51].

Finally, apart from inspecting the models’ robustness and predictivity, one should also define their applicability domain (AD), that is, the response and chemical structure space for which the models form reliable predictions without extrapolating. In this work, the AD of the developed models was determined by the leverage approach [52], which renders a measure of the similarity of a particular substance from all other substances (distance between its descriptor values and the average for all descriptor values). So, one can plot the standardized residuals against the leverage values for each DES of the several sets. From such a plot, the so-called William’s plot [52], we were able to identify the response and structural DES outliers. All plots shown in the present work were conceived with Matplotlib [53].

### 2.4. Consensus Modeling

The task here is to explore whether the overall quality of predictions for new substances might be improved by an “Intelligent” selection of multiple models. Towards that end, the most predictive QSPR models derived were subjected to consensus modeling, using the software tool Intelligent Consensus Predictor (freely available through the web https://dtclab.webs.com/software-tools, last accessed on 23 March 2022) developed by Roy et al. [54]. The four strategic techniques of this tool were applied, namely: Consensus Models (CM) 0–3, just as in our previous work [28], and as fully described in the work by the authors [54]. In short, CM0 is the simplest strategy and consists in computing the arithmetic average of predicted response values from all input individual models. In contrast, CM1 is based on the simple arithmetic average of predictions from all qualified individual models. CM2 corresponds to weighted average predictions from all qualified models, formerly giving appropriate weightage to those models. Finally, CM3 applies compound-wise predictions based on the best selection coming from the qualified models. Independently of the consensus modeling methodology, our main purpose was thus to combine multiple statistically robust models to improve the predictivity over the external validation set.

## 3. Results

### 3.1. Model Calibration and Evaluation

**Figure 1:**
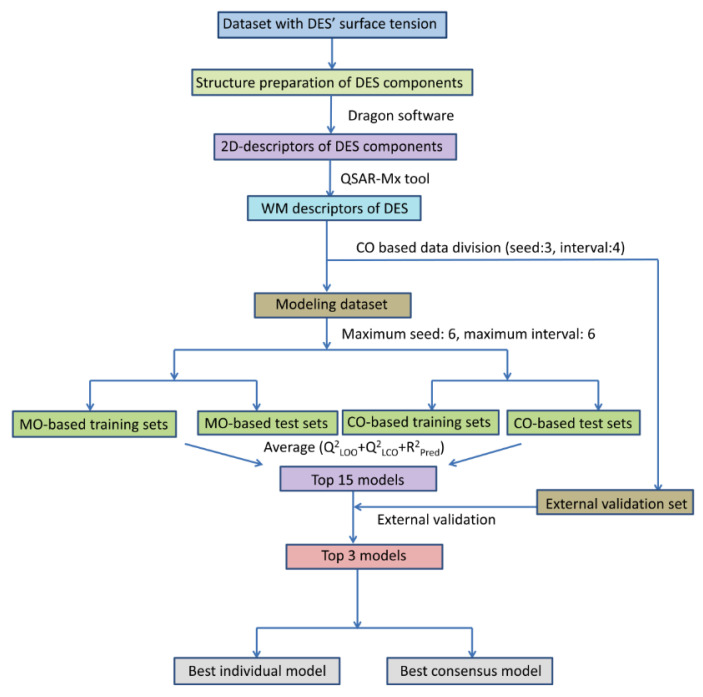
Basic workflow chart for the QSPR regression modeling, followed in this study.

Figure 1 depicts the workflow chart followed in the present QSPR modeling, which was mostly carried out using our recently developed tool, QSAR-Mx. As can be seen, all the involved steps and methodology employed to cope with the major goal of this work are shown, i.e., to build reliable predictive QSPR regression models from the compiled data that could be used to estimate the surface tension of DESs.

In total, 258 models were set up by varying data splitting schemes, descriptor calculation methods (Method-1 or Method-2) and SFS-MLR modeling. Among these, 136 models pertained to the MO-based division scheme, whereas the remaining 112 models were generated with the CO-based division scheme. The overall predictive quality of each of these regression models was judged by means of the average value computed for the statistical parameters *Q^2^*_LOO_, *Q*^2^_LCO_ and *R*^2^_Pred_. Essentially, the two parameters—*Q*^2^_LOO_ and *R*^2^_Pred_—account for the internal and external predictivity of the QSPR models, respectively. Nevertheless, the parameter *Q*^2^_LCO_ was also included to ensure that the most predictive models do not suffer overfitting due to bias towards some specific components of the binary DES mixtures. Naturally, the higher the average value obtained from these three parameters is, the more predictive the model is. Considering this, we selected the top 15 unique models for further processing. A summary of the statistical results of these models is given in Table 1. Interestingly, out of these 15 models, 14 were derived from MO-based data distributions, and only one model arose from CO-based distributions. Undoubtedly, that clearly shows that MO-based data distributions are more likely to produce more predictive models in comparison to the CO-based data distributions since the latter provides a more rigorous validation strategy.

As referred to before, in the entire model-building process, the data distributions were varied for the selection of the most predictive models. Therefore, the generated test sets serve as a validation set to estimate the external predictivity of the models but, at the same time, as calibration sets for the selection of the best models. In contrast, the external validation sets (containing 84 data points) were treated as the ‘true validation set’ for assessing the external predictivity of the models. The latter was built with the CO-based data-distribution scheme, thus holding a significant challenge to the generated models as far as their external predictivity is concerned. A comparison of the predictivity of the top 15 models is shown in Table 2.

As can be clearly observed from Table 2, only a few models show satisfactory predictions against the external validation set. Nevertheless, six of these models had R^2^_Pred_ values greater than 0.50, as well as average %AARD values lower than 12. Moreover, three models, namely M09, M10 and M12, supplied the most satisfactory predictivity towards such external validation set with *R*^2^_Pred_ > 0.65 and %AARD < 10. Therefore, these three models were considered the best models obtained for predicting the surface tension of DESs. Remarkably, M10, the only CO-based model included in the top 15, emerged as one of the most predictive models. Still, on the basis of overall predictivity, M12 was selected as the best individual QSPR model, even taking into account its slightly lower internal predictivity, compared to M10, and its slightly lower external predictivity towards the test set, as compared to M09. Even so, M12 afforded a balanced prediction against all three sets with an average %AARD value of 7.126, which is lower than that obtained for the other two models. At the same time, model M12 provides the best solution if the average value of *Q*^2^_LOO_, *Q*^2^_LCO_ and *R*^2^_Pred_ (against the two validation sets) is considered. In fact, for M12, this average value was found to be 0.859, while for M09 and M10, the average values were estimated as 0.820 and 0.831, respectively.

In summary, the best predictive model found for the DESs’ surface tension (a six-variable equation, model M12) can be expressed as detailed below, while the meaning of the selected WM descriptors is given in Table 3.

σ = +89.611 (±3.452)

+0.405 (±0.026) P_VSA_MR_6pmix

−5.034 (±0.874) Eig02_EA(dm)pmix

−23.145 (±3.320) CATS2D_02_ANpmix(3)

+8.835 (±0.174) BLTF96pmix

−25.191 (±2.352) MATS5snmix

−0.104 (±0.011) *T*

In this equation, *X*_pmix_ and X_nmix_ stand for WM descriptors of the type *D*_pmix_ in line with Equation (1) and *D*_nmix_ following Equation (2), respectively, *T* is the temperature (in K) under which the surface tension has been measured, and σ is the surface tension (in mN/m).

**Figure 2:**
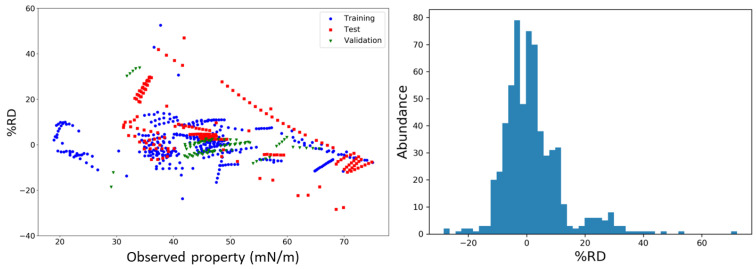
Relative deviations (%RD) between the predicted and observed DES surface tensions (**left**) and histogram plot of the distribution of %RD values (**right**).

A summary of the extended statistical results for model M12 is given in Table 4. The determination coefficient values (*R*^2^ = 0.916 and *R*^2^_Adj_ = 0.915), the sample size (*N*_tr_ = 360), the Fisher ratio (*F* = 642.4), but especially the high ratio between the number of data points to adjustable variables (*ρ* = 60) [59] are indicative of the model’s statistical significance and fitness. Model M12 also provides a satisfactory internal and external predictivity as follows from the cross-validation, *r_m_*^2^ and *R*^2^_Pred_ metrics values (see Table 4). Moreover, built with only six descriptors, it led to %AARD values of 5.805, 11.155 and 4.418 against the training, test and validation sets, respectively. The model prediction ability was further checked by analyzing the relative deviations (%RD = 100*(σ_Pred_ − σ_exp_)/σ_exp_) between the predicted and experimental DES surface tension values for all three sets. As Figure 2 shows, model M12 performs more accurately regarding the training and external validation sets than the test set. Yet, the latter also demonstrates a normal behavior considering the shape of the RD distribution according to the proposed model, also displayed in Figure 2. This histogram plot clearly depicts that most of the RD error values are within ±20% and that those are normally distributed, suggesting that the model estimations are not biased.

**Figure 3:**
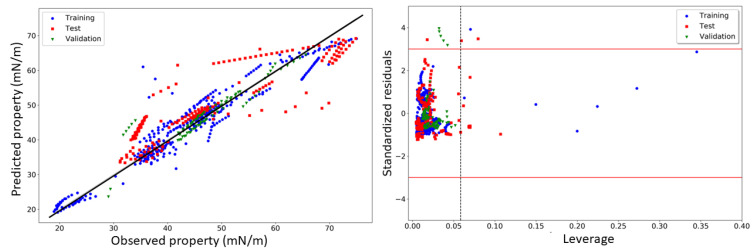
Predicted surface tension values vs. observed experimental ones (**left**) and Williams plot (**right**) obtained for model M12.

Figure 3 shows the plot of the predicted surface tensions obtained from model M12 vs. the observed experimental ones. As seen, the majority of the data points are sufficiently close to the diagonal line, denoting the model’s reliability and soundness of its predictions. Indeed, the model’s performance is even better than that of the previously developed thermodynamic model for the DES surface tension [14], which, despite having fewer data points (a total of 530 data points, considering only the 99 unique binary DES), led to %AARD values of 8.87 and 14.81 for the training and test sets, respectively. However, the purpose and outcomes of the current QSPR modeling are different from that of any thermodynamic model, as the former demands several different conditions to be satisfied, apart from validation, to establish the statistical robustness of the model. For example, so far, we have demonstrated the acceptable results on the reliability of the QSPR model M12, but it is also important to inspect the non-intercollinearity among any two of its descriptors. The latter was found to be 0.238, indicating that the variables included in the model are indeed not interrelated to each other. Furthermore, the model was itself checked for its uniqueness by the *Y*-based randomization technique, which was performed by scrambling the endpoint responses for the training set. The high value obtained for *cR_P_*^2^ (=0.908) implies that the model is not correlated by chance. Another crucial aspect is related to the applicability domain of the model that here was assessed by analyzing the Williams plot (plot of standardized residual vs. leverages). As seen in Figure 3, eight data points from the training set and thirteen from the test set can be considered structural outliers of the model, but no structural outliers were found in the external validation set. Interestingly, most of these structural outliers were well predicted by the model and were thus retained, as previously suggested by Gramatica et al. [49]. In addition, only twelve data points of the entire dataset were found to be response outliers, which also proves the high predictive accuracy of the model [60].

### 3.2. Model Interpretation

**Figure 4:**
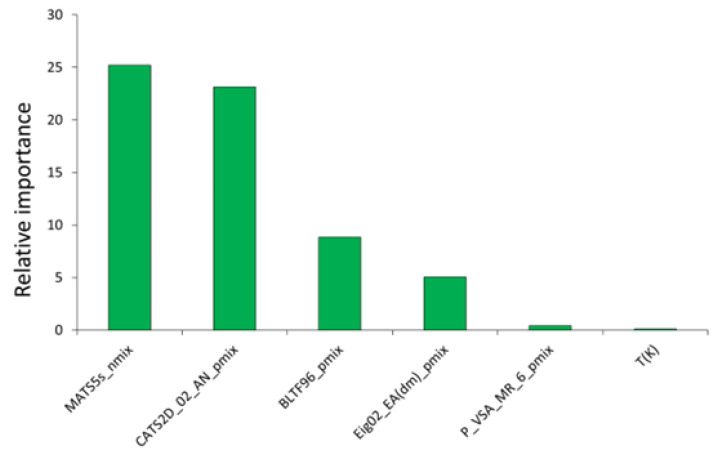
Relative importance of the descriptors found in the best individual model M12.

In our previous investigation on density [28] we observed that, in spite of providing less mechanistic interpretability, graph-based topological descriptors often help in characterizing the physicochemical properties. In the present work, a number of topological descriptors were also proven to be significant for describing the surface tension of DESs. Figure 4 shows the relative importance of each descriptor of model M12, estimated on the basis of the absolute value of its regression coefficients.

**Figure 5:**
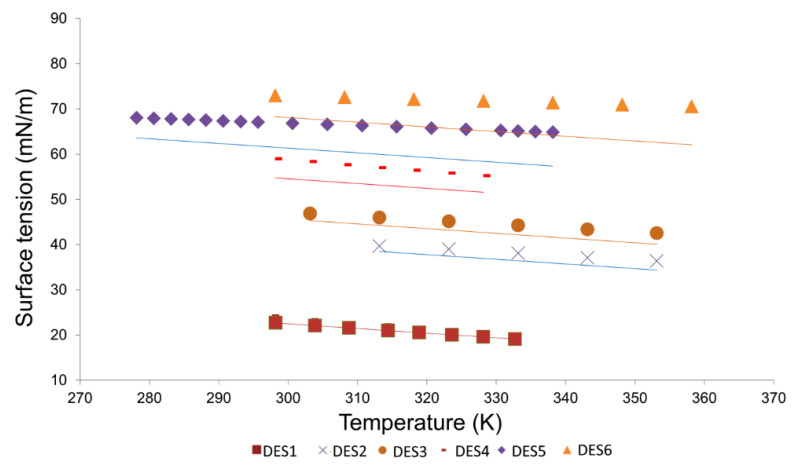
Comparison of surface tension calculated by the M12 model with literature data in the temperature range from 278.15-358.15 K for six DESs at atmospheric pressure. DES1: DL-menthol and octanoic acid (3:1); DES2: tetrabutylammonium chloride and arginine (8:1); DES3: tetraprpylammonium bromide and ethylene glycol (1:6); DES3: tetraprpylammonium bromide and ethylene glycol (1:6); DES4: N,N-diethylethanolammonium chloride and glycerol (1:5); DES5: choline chloride and glycerol (1:5); DES6: choline chloride and D-glucose (1:1).

As can be observed, the WM descriptor MATS5s_nmix_ was found to have the highest importance and besides, it is the only *D*_nmix_ type descriptor in the model. Being derived from graph-based topological descriptors, MATS5d_nmix_ points out that the differences in topological geometry of the DESs’ components may play a significant role in the surface tension of these solvents. The *D*_pmix_ type WM descriptor CATS2D_02_AN_pmix_ is the second most influencing descriptor of the model. Chemically Advanced Template Search (CATS) descriptors are a useful group of descriptors that account for the topological distance among scaffold features in the molecules [58]. CATS2D_02_AN, in particular, means that the acceptor and negatively charged groups are separated by a small topological distance (=2). In this case, higher values of this descriptor are found to be negatively correlated to the surface tension. Descriptor BLTF96_pmix_ appears as the third most important descriptor in the model. Unlike the first two descriptors of topological nature, this descriptor is based on an important molecular property—lipophilicity [55,56]. Since this descriptor belongs to the *D*_pmix_ type, it may be inferred that higher lipophilicity of the components would trigger higher surface tension for the DESs. Apart from lipophilicity, another well-known physicochemical property—dipole moment—was also found to have important contributions in ascertaining the DES surface tension. The importance of the dipole moment is derived from the presence of descriptor Eig02_EA(dm)_pmix_. The fifth most important descriptor belongs to the class of P_VSA descriptors, which represent the amount of van der Waals surface area (VSA) having a property (P) in a certain range [56]. In the case of the descriptor P_VSA_MR_6_pmix_, the property is the molar refractivity (MR) at a larger range (bin size 6). The positive relation of P_VSA_MR_6_pmix_ with the dependent property is highly significant as it suggests that increased MR (i.e., polarizability) within the van der Waals surface of each component contributes towards a higher surface tension for the respective DESs. Finally, the last descriptor of the model is the temperature of surface tension measurements, *T*. As expected, with increasing temperature the surface tension is found to decrease, which fits well with the experimental findings. Still, to further check how model M12 actually addresses the influence of temperature, we randomly selected six DESs with a range of surface tension values. From Figure 5, it can be clearly seen that both experimental and predicted properties followed the same trend, i.e., the surface tension gradually decreases as the temperature is increased.

### 3.3. Non-Linear Models

Albeit M12 emerged as the most accurate linear model in estimating the DES surface tension, the question that still remains is whether a non-linear model based on its descriptors might have better performance. Table 5 shows a statistical summary of the performance of the non-linear models resulting from applying the five different machine learning techniques, i.e.: *k*-NN, RF, SVM, NN-MLP and GB [49,50,51,52,53]. It can be observed that most of these ML techniques failed to produce predictive models and their results are not accurate either. Still, the SVM technique yields a predictive model and thus has the highest performance among the other ML techniques, though both the internal and the external predictivity of the latter remain inferior to the linear model. These results indicate that for the selected set of descriptors, the multiple linear regression-based model has the best accuracy in estimating the surface tension of DESs as well as sufficient predictivity that cannot be achieved with other model development techniques.

### 3.4. Consensus Modeling

Finally, we applied the intelligent consensus modeling [54] to see whether the surface tension predictions for the external validation set could be improved. To do so, sets of the three most predictive linear models—M09, M10 and M12—were subjected to consensus predictions in different combinations, namely: (a) C1-based using models M09, M10 and M12; (b) C2-based using models M10 and M12; (c) C3-based using models M09 and M10; and (d) C4-based with models M09 and M12. In each case, the modeling dataset containing 535 data points was treated as the training set whereas the external validation set was used to check the external predictivity of the consensus model. The results of all consensus modeling attempts are presented in Table 6.

Interestingly, the resulting models C1 and C4 lead to similar predictivities. Yet, none of the later consensus models display an external predictivity considerably better than that of the best individual model, M12. The *R*^2^_Pred_ and %AARD values obtained for consensus model C1 are 0.864 and 4.459, respectively, and similarly for C3 (i.e., 0.854 and 4.393). As can be seen, both C1 and C3 may therefore be projected as alternative models to M12. However, let us mainly focus on C3, since it reveals that M09 and M10 may indeed work as complementary models for each other towards improving the external predictivity. Details about the M09 and M10 models are provided in Table S3 of the SI.

Since M09 was developed with the same data distribution as M12, these two models have four descriptors in common, namely: CATS2D_02_AN_pmix_, P_VSA_MR_6_pmix_, BLTF96_pmix_, and *T*. Obviously, these four descriptors have a high significance in describing the surface tension of DESs. Significantly, CATS2D_02_AN_pmix_, which was found to be the second most important descriptor of M12, appears to be the most influential descriptor of M09. It undoubtedly indicates that this descriptor may be considered the most crucial descriptor in predicting the surface tension of DESs. Presumably, due to the similarity between M12 and M09, consensus modeling with these two models failed to provide any better solution. Model M10, in contrast, is established as a unique model because, save for *T*, none of its descriptors is found either in M09 or in M12. Most likely due to this reason, its combination with the other two models produces good consensus models. Unlike models M09 and M12, model M10 yields are slightly higher but still have acceptable intercollinearity between descriptors, with a maximum *R*^2^ value of 0.713. The selected descriptors for models M09 and M10 are described in detail in Table S4 of the SI.

## 4. Conclusions

The present work aimed to establish a systematic and well-designed QSPR modeling for predicting the surface tension of a wide range of DESs, following the OECD guidelines. Towards such aim, the largest surface tension data bank of binary DESs known to date, comprising 619 data points from 113 unique DESs of various families was gathered from the literature. In addition, special emphasis was put on employing robust validation strategies for setting up the QSPR models. In so doing, the best QSPR models were set up with multiple data distributions resulting from MO- and CO-data splitting schemes along with a weighted mixture type of descriptors, using our in-house open access tool QSAR-Mx. After considering several statistical parameters, the top three individual linear regression models stand out for their accuracy and robustness. The most predictive individual linear model was however selected based on the predictivity towards the external validation set.

Similar to our previous study on the development of a thermodynamic model [14], the surface tension of DESs was found to be a particularly difficult property to predict. This may be related to the challenging nature of the accurate experimental estimation of surface tensions, associated with the presence of surface-active impurities and differences in the measuring protocols [33]. Nevertheless, our most predictive individual QSPR regression model (i.e., model M12) yielded a satisfactory overall %AARD value (=7.126), especially when compared to the aforementioned thermodynamic model (%AARD = 10.31, considering only the binary DESs therein) [14]. This model depicted also the structural and physicochemical features related to the surface tension of DESs. Just as in our previously developed model for the density of DESs [28], graph-based topological descriptors were found to be highly useful in this respect. Some physicochemical factors, such as the lipophilicity, polarizability and dipole moment of the DESs’ components, were found to be responsible for ruling their surface tensions. We also attempted to generate consensus models based on the top three individual linear models. Interestingly, consensus models based on the two other best individual models—M09 and M10—were found to be equally predictive towards the external validation set.

Overall, this work definitely provides valuable information about the structural and physicochemical features required for predicting the surface tension of binary DESs. At the same time, it also lends important guidelines to set up predictive and validated linear interpretable QSPR models for the various properties of binary mixtures. The high predictivity of the models ensures that these models may be used on the industrial scale to at least predict the surface tension of the DESs that are newly developed or under-developed to assess their suitability as an industrial solvent. The models may also be used to screen a large number of DESs (obtained from databases) to predict the DESs with desirable surface tension properties. What is more, all the proposed models are easily reproducible since they rely on fully specified computational procedures and were built with non-commercial software tools. Finally, both the individual and consensus models developed in this work shall help the future screening as well as the design of new sustainable DES, with major time and cost savings.

## Figures and Tables

**Table 1:** Statistical results of the top 15 unique QSPR regression models generated.

Model	Seed; Interval	Descriptor ^a^	Split ^b^	Scoring ^c^	*N*_tr_^d^	*Q* ^2^_LOO_^e^	*Q* ^2^_LCO_^f^	MAE_LOO_^g^	*N*_ts_^h^	*R* ^2^_Pred_^i^	MAE_test_^j^	Avg ^k^
M01	2; 5	Method-1	MO	NMAE	408	0.884	0.854	2.586	127	0.907	3.863	0.882
M02	4; 5	Method-2	MO	NMAE	435	0.873	0.849	2.658	100	0.899	3.966	0.874
M03	1; 4	Method-2	MO	NMAE	408	0.898	0.854	2.775	127	0.865	2.569	0.872
M04	5; 4	Method-2	MO	NMAE	409	0.898	0.855	2.771	126	0.862	2.584	0.872
M05	4; 5	Method-1	MO	NMAE	435	0.871	0.839	2.635	100	0.898	4.039	0.869
M06	3; 5	Method-1	MO	NMAE	443	0.881	0.849	2.671	92	0.871	4.073	0.867
M07	1; 3	Method-1	MO	NMAE	359	0.901	0.862	2.369	176	0.836	4.223	0.866
M08	4; 5	Method-1	MO	R^2^	435	0.883	0.858	2.854	100	0.855	4.695	0.865
M09	4; 3	Method-1	MO	NMAE	360	0.906	0.854	2.322	175	0.83	4.389	0.864
M10	1; 2	Method-2	CO	R^2^	301	0.931	0.903	1.660	234	0.754	6.030	0.862
M11	4; 5	Method-2	MO	5-fold	435	0.865	0.841	3.000	100	0.876	4.282	0.861
M12	4; 3	Method-2	MO	R^2^	360	0.908	0.882	2.608	175	0.783	5.134	0.858
M13	4; 5	Method-1	MO	5-fold	435	0.871	0.845	2.706	100	0.857	4.626	0.858
M14	1; 4	Method-1	MO	10-fold	408	0.869	0.849	3.340	127	0.847	2.050	0.855
M15	5; 4	Method-1	MO	10-fold	409	0.869	0.85	3.336	126	0.844	2.052	0.855

**^a^** Descriptor calculation method used. **^b^** Data splitting scheme utilized. **^c^** Scoring condition applied. **^d^** Number of data points in the training set. **^e^** Leave-one-out cross-validation determination coefficient. **^f^** Leave-chemical-out cross-validation determination coefficient. **^g^** LOO cross-validation mean absolute error. **^h^** Number of data points in the test set. **^i^** Variance explained for external prediction. **^j^** Mean absolute error of the test set. **^k^** Average value of *Q*^2^_LOO_, *Q*^2^_LCO_ and *R*^2^_Pred_.

**Table 2:** Internal and external predictivity for the top 15 regression models against the training, test and external validation sets **^a^**.

Model ^b^	Training Set				Test Set			External Validation Set		
	*N*_tr_	*Q* ^2^_LOO_	*Q* ^2^_LCO_	%AARD	*N*_ts_	*R* ^2^_Pred_	%AARD	*N*_ex_^c^	*R* ^2^_Pred_	%AARD
M01	408	0.884	0.854	5.541	127	0.907	11.843	84	−0.335	15.931
M02	435	0.873	0.849	6.11	100	0.899	7.517	84	0.464	12.176
M03	408	0.898	0.854	6.063	127	0.865	5.418	84	−0.196	15.833
M04	409	0.898	0.855	6.057	126	0.862	5.446	84	−0.19	15.818
M05	435	0.871	0.839	5.965	100	0.898	7.538	84	0.392	11.838
M06	443	0.881	0.849	6.052	92	0.871	7.65	84	0.516	11.021
M07	359	0.901	0.862	5.222	176	0.836	9.331	84	−7.225	27.7
M08	435	0.883	0.858	6.6	100	0.855	8.456	84	0.466	11.45
**M09**	**360**	**0.906**	**0.854**	**5.202**	**175**	**0.83**	**9.872**	**84**	**0.688**	**8.527**
**M10**	**301**	**0.931**	**0.903**	**4.208**	**234**	**0.754**	**12.754**	**84**	**0.734**	**7.777**
M11	435	0.865	0.841	6.875	100	0.876	7.78	84	0.568	10.047
**M12**	**360**	**0.908**	**0.882**	**5.805**	**175**	**0.783**	**11.155**	**84**	**0.862**	**4.418**
M13	435	0.871	0.845	6.204	100	0.857	8.442	84	0.544	11.093
M14	408	0.869	0.849	7.344	127	0.847	3.943	84	0.352	11.642
M15	409	0.869	0.85	7.339	126	0.844	3.929	84	0.353	11.638

**^a^** For the meaning of *N*_tr_, *N*_ts_, *Q*^2^_LOO_, *Q*^2^_LCO_, *R*^2^_Pred_ and %AARD, please check the footnotes of Table 1. **^b^** The more predictive models are marked in bold. **^c^** Number of data points in the external validation set.

**Table 3:** The five WM molecular descriptors selected for model M12—Equation (3).

Symbol	Definition [55,56,57,58]	Class
P_VSA_MR_6_pmix_	P_VSA-like on Molar Refractivity, at bin size 6	P_VSA-like descriptor ^**a**^ (*D*_pmix_ type)
Eig02_EA(dm)_pmix_	eigenvalue n. 2 from edge adjacency matrix, weighted by dipole moment	Edge adjacency indices (*D*_pmix_ type)
CATS2D_02_AN_pmix_	CATS2D Acceptor-Negative at lag 2	2D CATS ^**b**^ (*D*_pmix_ type)
BLTF96_pmix_	Verhaar Fish base-line toxicity from MLOGP (mmol/L)	Molecular properties (*D*_pmix_ type)
MATS5s_nmix_	Moran autocorrelation of lag 5, weighted by I-state ^**c**^	2D autocorrelations (*D*_nmix_ type)

**^a^** P_VSA-like descriptors stand for the van der Waals surface area (VSA) with a particular property (P), in this case, the molar refractivity (MR) [57]. **^b^** Chemically Advanced Template Search (CATS) descriptors expressly designed to identify scaffold hops [58]. **^c^** I-states are based on the Kier-Hall atomic electronegativity modified by the number of σ bonds, number of hydrogen atoms, number of electrons in π orbitals, and number of lone pair electrons [55,56].

**Table 4:** MLR statistical results for model M12—Equation (3) **^a^**.

Training Set	Test Set	External Set
*N*_tr_ = 360;	*N*_ts_ = 175;	*N*_ex_ = 84;
*R*^2^ = 0.916; *R*^2^_Adj_ = 0.915; *F*(6353) = 642.4;	*R*^2^_Pred_ = 0.783;	*R*^2^_Pred_ = 0.862;
*Q*^2^_LOO_ = 0.908; MAE_LOO_ = 2.608; *Q*^2^_LCO_ = 0.882; MAE_LCO_ = 3.122;	MAE = 5.134;	MAE = 1.777;
*r*_m_^2^_(LOO)_ = 0.869, ∆*r_m_*^2^_(LOO)_ = 0.066;	*r_m_*^2^_(test)_ = 0.573, ∆*r_m_*^2^_(test)_ = 0.197;	*r_m_*^2^_(ext)_ = 0.767, ∆*r_m_*^2^_(ext)_ = 0.097;
%AARD = 5.805; *^c^R_P_*^2^ (1000 runs) = 0.908	%AARD = 11.155	%AARD = 4.418

**^a^** *R*^2^: Determination coefficient; *R*^2^_Adj_: Adjusted *R*^2^; *F*(6,353): Fisher’s statistic; MAE_LOO_ and MAE_LCO_: Leave-one-out and leave-chemicals-out cross-validation mean absolute error, respectively; *r_m_*^2^_(LOO)_ and ∆*r_m_*^2^_(LOO)_: LOO cross-validation *r_m_*^2^ and its associated deviation, respectively; *r_m_*^2^_(test)_ and ∆*r_m_*^2^_(test)_: *r_m_*^2^ of the test set and its associated deviation, respectively; *r_m_*^2^_(ext)_ and ∆*r_m_*^2^_(ext)_: *r_m_*^2^ of the external test set and its associated deviation, respectively. For the meaning of *N*_tr_, *N*_ts_, *N*_ex_, *Q*^2^_LOO_, *Q*^2^_LCO_, *R*^2^_Pred_, and %AARD, check the footnotes of Table 1 and Table 2.

**Table 5:** Summary of the statistical parameters obtained from non-linear models based on different machine learning methods.

Method ^a^	Training Set (*Q*^2^_5-fold_)	Test Set (*R*^2^_Pred_)	External Set (*R*^2^_Pred_)
*k*-NN	0.176	0.597	not determined
RF	0.473	0.746	not determined
SVM	0.874	0.774	0.767
MLP	0.541	0.269	not determined
GB	0.453	0.471	not determined

^a^ *k*-NN: *k*-Nearest Neighbors; RF: Random Forests; SVM: Support Vector Machines; NN-MLP: Neural Network Multilayer Perceptron; GB: Gradient boosting.

**Table 6:** External predictivity of the best individual model M12 and consensus models (C1-C4) built with different combinations of the top three models (M09, M10 and M12).

Consensus Models	Models	CM ^a^	*R* ^2^_Pred_^b^	*r_m_* ^2^_(test)_^c^	MAE_test_^d^	%AARD ^e^
C1	M09, M10, M12	0	0.864	0.801	1.869	4.459
C2	M10 and M12	2	0.823	0.812	2.089	4.732
C3	M09 and M10	2	0.853	0.787	1.979	4.393
C4	M09 and M12	None	-----	-----	-----	-----
M12	-----	-----	0.862	0.767	1.777	4.418

**^a^** Method of Intelligent consensus prediction that yielded the best external validation result. **^b^** Variance explained for the external prediction. **^c^** Metric *r_m_*^2^ for the test set. **^d^** Mean absolute error for the test set. **^e^** Absolute average relative deviation.

## Data Availability

All the data files pertaining to the QSPR modeling are available from the authors. The training, test and external datasets were taken from cited publications, and the DES chemical structures along with the collected surface tension data are provided in the Supporting Information (Table S1). Dragon 7.0, MarvinView, and Standardizer were used in this study under academic license (see Material and methods section). Three other open source software tools were also used in this study, namely: QSAR-Mx, a Python-based tool developed by the authors that is available to download at https://github.com/ncordeirfcup/QSAR-Mx (last accessed on 28 April 2022); Mlxtend, a Python library of useful tools that is accessible from https://rasbt.github.io/mlxtend/; scikit-learn, a Python library of useful machine learning tools that is accessible from https://scikit-learn.org/stable/; and Intelligent Consensus Predictor, a Java-based tool available through the web https://dtclab.webs.com/software-tools (last accessed on 23 March 2022)(see Material and methods section).

## Supplementary Materials

The following supporting information can be downloaded at: https://www.mdpi.com/article/10.3390/molecules27154896/s1, Table S1. List of all investigated DESs and experimental surface tension data. (XLSX); Table S2. Hyperparameter tuning of the different machine learning techniques. (PDF); Table S3. Detailed description of the M09 and M10 models. (PDF); Table S4. Descriptors used in the M09 and M10 models. (PDF)

## Sample Availability

Samples of the compounds (full dataset used for modeling) are available from the authors.
