# Supplementary material for: Predicting the Surface Tension of Deep Eutectic Solvents: A Step Forward in the Use of Greener Solvents
Source: Molecules. 2022 Jul 31;27(15):4896. doi: 10.3390/molecules27154896 (PMC9370217; doi:10.3390/molecules27154896)
Supplement: Supplementary file 1 [file molecules-27-04896-s001.zip › molecules-1835999-supplementary/TablesS2_S4.pdf]

Supporting Information

Predicting the Surface Tension of Binary Deep Eutectic Solvents: A Step forward in the Use of Greener Solvents

Amit Kumar Halder[1,2*], Reza Haghbakhsh[3,4], Iuliia V. Voroshylova[1], Ana Rita C. Duarte[4], M. Natália D. S. Cordeiro[1*]

[1]LAQV@REQUIMTE/Department of Chemistry and Biochemistry, Faculty of Sciences, University of Porto, 4169-007 Porto, Portugal

[2]Dr. B. C. Roy College of Pharmacy and Allied Health Sciences, Dr. Meghnad Saha Sarani, Bidhannagar, Durgapur 713212, West Bengal, India

[3]Department of Chemical Engineering, Faculty of Engineering, University of Isfahan, 81746-73441, Isfahan, Iran

[4]LAQV@REQUIMTE/Department of Chemistry, NOVA School of Science and Technology, 2829-516 Caparica, Portugal

* Corresponding authors: amit.halder@f.up.pt; ncordeir@fc.up.pt

Table S2. Hyperparameter tuning of the different machine learning techniques. [a]

Technique	Parameters varied
RF	Bootstrap: True/ False<br>Criterion: MSE, MAE<br>Maximum depth: 10, 30, 50, 70, 90, 100, 200, None<br>Maximum features: Auto, Sqrt, Log2<br>Minimum samples leaf: 1, 2, 4<br>Minimum samples split: 2, 5, 10<br>Number of estimators: 50, 100, 200
k-NN	Number of neighbours: 1-50<br>Weight options: Uniform, Distance<br>Algorithms: Auto, Ball Tree, KD Tree, Brute
SVR	C: 0.1, 1, 10, 100, 1000<br>Gamma: 1, 0.1, 0.01, 0.001<br>Kernel: RBF, Linear
NN-MLP	Hidden layer sizes: (10,)/(50,)/(100,)<br>Activation: Identity, Logistic, Tanh, Relu<br>Alpha: 0.0001, 0.001, 0.01, 1<br>Learning rate: Constant, Adaptive, Invscaling
GB	Loss: ls, lad<br>Learning rate: 0.01, 0.05, 0.1, 0.2<br>Min samples split: 0.1,0.2,0.3,0.4,0.5<br>Minimum samples leaf: 0.1,0.2,0.3,0.4,0.5<br>Maximum depth: 3,5,8<br>Maximum features: Log2, Sqrt<br>Criterion: Friedman MSE, MAE<br>Subsample: 0.5, 0.6, 0.8<br>Number of estimators: 50, 100, 200, 300

[a] Wherever applicable, a random seed value of 42 was chosen.

Table S3. Detailed description of the M09 and M10 models along with their statistical results.

Model	Equation	Training set	Test set	External validation set
	σ = +85.323 (±3.677)	N_{tr} = 360; R^2 = 0.916;	N_{ts} = 175;	N_{ex} = 84;
	+ 0.313 (±0.029) P_VSA_MR_6$_{pmix}$	R^2_{Adj} = 0.914;	R^2_{Pred} = 0.830;	R^2_{Pred} = 0.688;
	−3.009 (±0.747) VE3sign_B(m)$_{pmix}$	$F(7,352)$ = 547.7;	MAE =5.134,	MAE =3.942,
M09	+15.516 (±2.721) VE1sign_B(s)$_{pmix}$	Q^2_{LOO} =0.906; MAE$_{LOO}$ = 2.322;	r_m^2 $_{(test)}$ = 0.604;	r_m^2 $_{(test)}$ =0.704;
	+25.094(±3.319) nRNHO$_{pmix}$	Q^2_{LCO} =0.854; MAE$_{LCO}$ = 3.146;	Δr_m^2 $_{(test)}$ = 0.171;	Δr_m^2 $_{(test)}$ =0.154;
	−37.152 (±2.957) CATS2D_02_AN$_{pmix}$	$r_m^2$$_{(LOO)}$ = 0.866; $\Delta r_m^2$$_{(LOO)}$ = 0.064;	%AARD = 9.872	%AARD = 8.527
	+ 7.765 (±0.221) BLTF96$_{pmix}$	%AARD = 5.202;		
	−0.121 (±0.011) T(K)	cR_P^2 (1000 runs)= 0.906		
	σ = +79.350 (±2.661)	N_{tr} = 301; R^2 = 0.938;	N_{ts} = 234;	N_{ex} = 84;
	+6.681 (±0.253) Qindex$_{pmix}$	R^2_{Adj} = 0.937;	R^2_{Pred} = 0.753;	R^2_{Pred} = 0.734;
	+16.749 (±1.617) MATS4m$_{pmix}$	$F(5,295)$ = 895.3;	MAE =5.134,	MAE = 3.800,
M10	−6.429 (±0.125) CATS2D_02_LL$_{pmix}$	Q^2_{LOO} =0.931; MAE$_{LOO}$ = 1.660;	r_m^2 $_{(test)}$ = 0.458;	r_m^2 $_{(test)}$ = 0.742;
	+69.566 (±4.648) VE1sign_Dz(p)$_{nmix}$	Q^2_{LCO} =0.903; MAE$_{LCO}$ = 1.967;	Δr_m^2 $_{(test)}$ = 0.165;	Δr_m^2 $_{(test)}$ = 0.149;
	−0.117 (±0.008) T(K)	$r_m^2$$_{(LOO)}$ = 0.901; $\Delta r_m^2$$_{(LOO)}$ = 0.048;	%AARD = 12.754	%AARD = 7.777
		%AARD = 4.208;		
		cR_P^2 (1000 runs)= 0.916		

Table S4. Meaning of the descriptors used in the M09 and M10 models [1,2].

WM Descriptor	Core Descriptor	Definition	Class
VE3sign_B(m)$_{pmix}$	VE3sign_B(m)	Logarithmic coefficient sum of the last eigenvector from Burden matrix weighted by mass	2D matrix-based descriptors (D_{pmix} type)
VE1sign_B(s)$_{pmix}$	VE1sign_B(s)	Coefficient sum of the last eigenvector from Burden matrix weighted by I-State	2D matrix-based descriptors (D_{pmix} type)
nRNHO$_{pmix}$	nRNHO	Number of hydroxylamines (aliphatic)	Functional group counts (D_{pmix} type)
Qindex$_{pmix}$	Qindex	Quadratic index	Topological indices (D_{pmix} type)
MATS4m$_{pmix}$	MATS4m	Moran autocorrelation of lag 4 weighted by mass	2D autocorrelations (D_{pmix} type)
VE1sign_Dz(p)$_{nmix}$	VE1sign_Dz(p)	Coefficient sum of the last eigenvector from Barysz matrix weighted by polarizability	2D matrix-based descriptors (D_{pmix} type)
CATS2D_02_LL$_{pmix}$	CATS2D_02_LL	CATS2D Lipophilic-Lipophilic at lag 02	2D CATS counts (D_{pmix} type)

References

(1) Todeschini, R.; Consonni, V. Handbook of Molecular Descriptors; Wiley-VCH: Weinheim, Germany, 2000.

(2) Todeschini, R.; Consonni, V. Molecular Descriptors for Chemoinformatics, 2nd ed., Wiley-VCH, Weinheim, Germany, 2009.
